# Nutrient and Food Group Prediction as Orchestrated by an Automated Image Recognition System in a Smartphone App (CALO mama): Validation Study

**DOI:** 10.2196/31875

**Published:** 2022-01-10

**Authors:** Yuki Sasaki, Koryu Sato, Satomi Kobayashi, Keiko Asakura

**Affiliations:** 1 Link & Communication Inc Tokyo Japan; 2 Department of Social Epidemiology, Graduate School of Medicine and School of Public Health Kyoto University Kyoto Japan; 3 Department of Environmental and Occupational Health, School of Medicine Toho University Tokyo Japan

**Keywords:** health app, image recognition, automatic calculation, nutrient and food contents, validity, mobile phone, mHealth, validation, nutrition, diet, food

## Abstract

**Background:**

A smartphone image recognition app is expected to be a novel tool for measuring nutrients and food intake, but its performance has not been well evaluated.

**Objective:**

We assessed the accuracy of the performance of an image recognition app called *CALO mama* in terms of the nutrient and food group contents automatically estimated by the app.

**Methods:**

We prepared 120 meal samples for which the nutrients and food groups were calculated. Next, we predicted the nutrients and food groups included in the meals from their photographs by using (1) automated image recognition only and (2) manual modification after automatic identification.

**Results:**

Predictions generated using only image recognition were similar to the actual data on the weight of meals and were accurate for 11 out of 30 nutrients and 4 out of 15 food groups. The app underestimated energy, 19 nutrients, and 9 food groups, while it overestimated dairy products and confectioneries. After manual modification, the predictions were similar for energy, accurately capturing the nutrients for 29 out of 30 of meals and the food groups for 10 out of 15 meals. The app underestimated pulses, fruits, and meats, while it overestimated weight, vitamin C, vegetables, and confectioneries.

**Conclusions:**

The results of this study suggest that manual modification after prediction using image recognition improves the performance of the app in assessing the nutrients and food groups of meals. Our findings suggest that image recognition has the potential to achieve a description of the dietary intakes of populations by using “precision nutrition” (a comprehensive and dynamic approach to developing tailored nutritional recommendations) for individuals.

## Introduction

Noncommunicable diseases such as cardiovascular disease, diabetes, and some forms of cancer pose severe public health problems and constitute serious global social welfare issues [1]. Diet is a key modifiable factor related to health [1,2]. National strategies to improve diet and physical activity patterns at the population level have been implemented worldwide [1], and Japan has a national healthy diet goal for improving public health [3]. To plan and evaluate the achievement of these goals, the development of effective and adequate dietary assessment tools at the population level has long been a focus in the public health field.

In the research field, dietary recalls, dietary records, and food frequency questionnaires have been widely used as general dietary assessment tools [4]. Each of these methods has its strengths and limitations such as placing a burden on participants and researchers or limited accuracy. These tools are strongly dependent on researcher training and competency for dietary recalls, participant literacy, high motivation to maintain dietary records, and participant literacy and memory for questionnaires. New dietary assessment tools that utilize computers, internet, telecommunications, and imaging analysis technology have been developed and advanced [5]. Some of these new tools do not involve self-reporting or a dietitian’s entry of data, but instead employ automated image recognition for food photographs taken by a smartphone and an automated calculation system of nutrient intake [6-11]. Some of these tools provide automatic feedback on the nutrient intake of individuals, which may improve dietary outcomes and promote behavioral changes among users [6,12]. Advancements in these new technological tools will allow users to monitor their daily dietary habits and enable researchers to assess dietary intake at the population level more easily than ever before.

These comprehensive dietary assessment tools, which use image recognition systems to evaluate nutrient and food contents, have not yet had their validity experimentally evaluated. Previous experimental studies have examined the validity of the estimated quantities of energy and protein [11] or carbohydrates [13] in samples assessed using new dietary assessment tools, and other studies have examined the validity of food portion sizes [14,15]. In the epidemiological field, some validation studies have examined the validity of these apps but only for energy intake [16,17]. Two studies have evaluated the validity of these apps in assessing selected nutrient intakes [18,19], and 1 validation study has examined efficacy at monitoring the intake of the 4 food groups [20]. These new dietary assessment tools have already been used in many weight-loss intervention studies [21], and yet, their validity regarding comprehensive nutrients and food groups has not yet been assessed.

In this study, we examined the validity of nutrient and food group content assessed using an image recognition app called *CALO mama*. This study explored the potential of a smartphone app to estimate dietary intake at the population level in daily life.

## Methods

### A Smartphone App With an Image Recognition System

Link & Communication Inc (Tokyo, Japan) recently developed a health app for smartphones called *CALO mama*. Users of *CALO mama* can register their diet, exercise, mood, and quality of sleep on a daily basis. *CALO mama* offers the automated image recognition of meals and can automatically calculate nutrient and food content based on photographs taken by users. Additionally, artificial intelligence creates specific dietary recommendations for users based on their registered meals. For example, it warns users with nutrient deficiencies or an excess intake of fat, sugar, and salt, and indicates what they ought to watch out for in their next meal.

**Figure 1:**
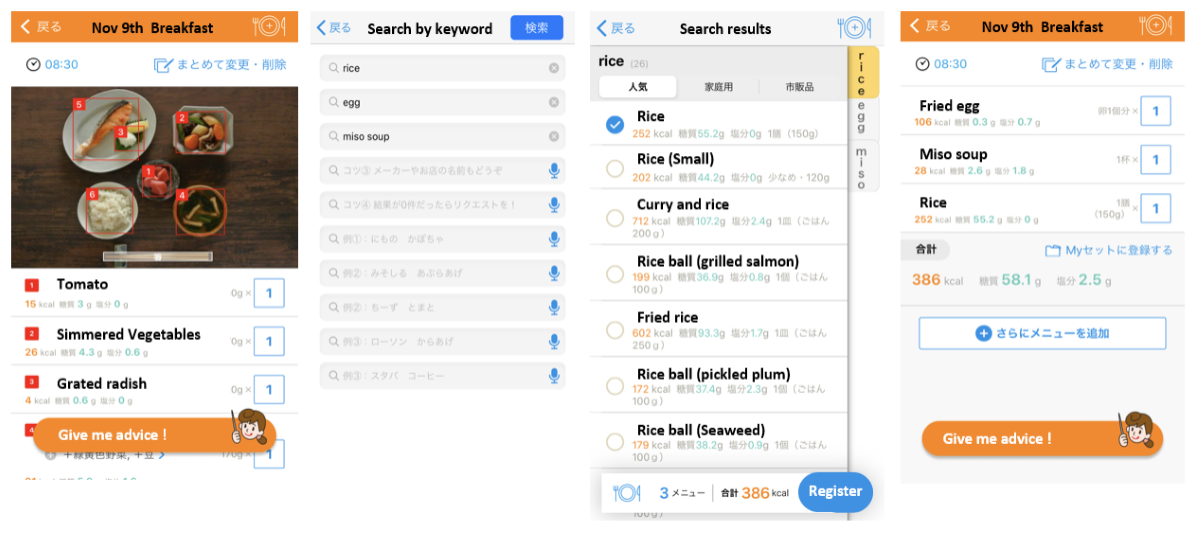
Screenshots of meal recording using the *CALO mama* app.

Figure 1 shows screenshots of this app illustrating how to identify and record meals. This app has a built-in list of approximately 150,000 food items, including fresh food, self-made meals, ready meals, and commercial products. Nationally registered dietitians developed a list of food items by referring to several recipe books and their standard energy and nutrient contents based on the Standard Tables of Food Composition in Japan [22]. Ready meals and commercial products were also registered from approximately 450 manufacturers and restaurants by dietitians. First, photographs of the meals taken by users are sent to a cloud server. An automated image recognition system involving deep learning predicts food items from a list of the standard 215 items and identifies ingredients in each item and portion size. Next, another system calculates the nutritional values of the items based on the predictions made by the image recognition system. Finally, the predicted names of the items, their portion sizes, and corresponding nutrition values are displayed on users’ smartphones. If the outputs appear imprecise, users can manually search for appropriate food items from the full list of approximately 150,000 items, modify the name and portion size of each item, and record them. For example, automated image recognition can distinguish coffee with milk and without milk based on the color of the liquid. In contrast, it is difficult to detect the difference between foods that cannot be determined from their external shape or color. If, for example, the app imprecisely recognizes diet coke and low-fat yogurt as a sugar-sweetened beverage and full-fat yogurt, respectively, based on the list of the standard 215 items, users can select the correct items from the full list and modify their records.

### Study Protocol

**Figure 2:**
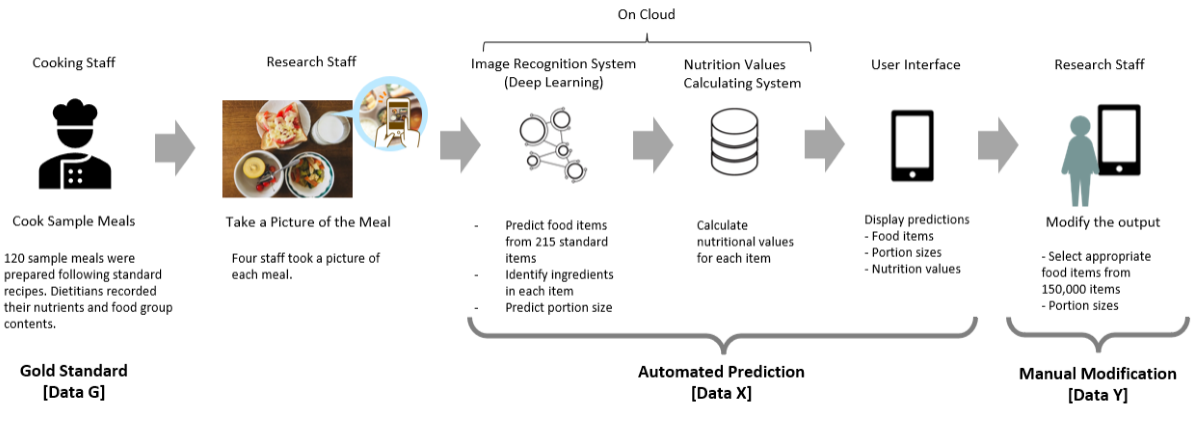
The study protocol using the *CALO mama* app.

Figure 2 shows how the results of our study data were obtained. A total of 120 sample meals, including 3-9 food dishes, were prepared by cooking staff (Table 1); 97 out of 120 (80.8%) sample meals had 4-6 dishes. Basically, the sample meals were cooked following standard recipes, but the cooking staff were allowed to remove or add ingredients while cooking. Dietitians observed the cooking process and recorded the nutrient and food group contents of the 120 sample meals as the gold standard (data G).

**Table 1:** The number of dishes included in a sample meal (N=120 sample meals).^a^

Dishes (n)	Sample meals, n (%)
3	12 (10)
4	35 (29.2)
5	44 (36.7)
6	18 (15)
7	9 (7.5)
8	1 (0.8)
9	1 (0.8)

^a^“Dishes” means dishes including multiple ingredients and foods such as a glass of milk or an apple, which can be regarded as 1 item in a meal.

The data of the 120 sample meals were registered in the app by 20 research staff who were recruited by Link & Communication Inc and blinded to the cooking process. First, the research staff took photographs of the sample meals and uploaded them to the app. Subsequently, we obtained the data regarding the nutrition and food content of the meals automatically predicted by the app (data X). In the next step, the staff were allowed to manually modify the name and portion size of each item based on their visual inspection. If the staff found that some ingredients needed to be added or removed, they modified the outputs from the app and recorded them (data Y). For every sample meal, 4 research staff registered data X and Y. We calculated the means of the 4 entries of data X and Y and compared them with data G as the gold standard.

### Statistical Analysis

Weight, energy, nutrient, and food group contents derived from data G, X, and Y are presented as means and standard deviations. The food groups used in this study are shown in Table 2. The means of the difference between data X or Y and data G were also calculated. Statistically significant differences between data X or Y and data G in each of the dietary variables were determined with the paired *t* test using 2-sided values. Statistical significance was set at *P*<.05. Further, we evaluated the agreement for the energy and macronutrients of data X and Y compared with data G by using Bland-Altman plots [23]. All statistical analyses were conducted using the SPSS statistical software package version 36 (IBM Corp).

**Table 2:** Definition of the food groups.

Food group	Item number in the Standard Tables of Food Composition in Japan [22] and definition^a^
Cereals	1001-1166
Potatoes	2001, 2006-2027, 2041, 2045-2055
Pulses	4001-4094 and % energy of carbohydrate <51%
Nuts	5001-5037 and dietary fiber >6.1 g/100 g and polyunsaturated fat >12 g/100 g
Vegetables	6001-6362
Green and yellow vegetables	6001-6362 and β-carotene ≥600 μg/100 g, or 6182, 6007-6011, 6020, 6021, 6093, 6094, 6157, 6158, 6245, 6246, 6237
Fruits	7001-7176 and carbohydrate <39 g, salt=0 g, and not included beverages, canned, or preserved in syrup
Mushrooms	8001-8052
Seaweed	9001-9055, Korean-style laver (ie, dried, edible seaweed)
Fish and shellfish	10001-10362, 10389-10423
Meats	11001-11197, 11199-11293
Eggs	12020-12016, 12020
Dairy products	13001-13041, 13048-13058 and calcium ≥100 mg/100 g
Confectioneries	15001-15141
Alcoholic beverages	16001-16024, 16027-16032, tequila, liqueur, *shochu* mixed with carbonated beverage, *makgeolli*

^a^Food items not given an item number in the Standard Tables of Food Composition in Japan are described with food names.

## Results

The means and standard deviations of meal weight and energy, nutrients, and food group contents derived from data G, X, and Y are shown in Table 3. Data X were similar to data G in weight, accurately capturing 11 out of 30 nutrients and 4 out of 15 food groups; it underestimated energy, capturing only 19 nutrients and 9 food groups, while it overestimated dairy products and confectioneries. After manual modification, data Y were similar to data G in energy, accurately capturing 29 out of 30 nutrients and 10 out of 15 food groups; it underestimated pulses, fruits, and meats, while it overestimated weight, vitamin C, vegetables, and confectioneries.

**Figure 3:**
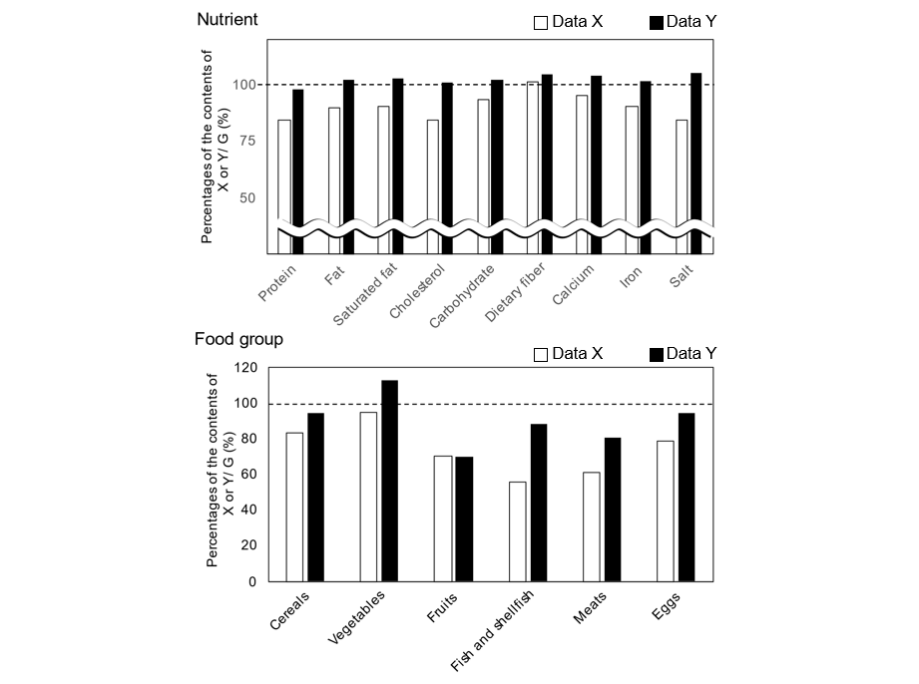
Proportions of the selected nutrient and food group contents of automatically recognized meals (data X) and manually adjusted meals (data Y) from *CALO mama* to the gold standard of sample meals (data G).

**Figure 4:**
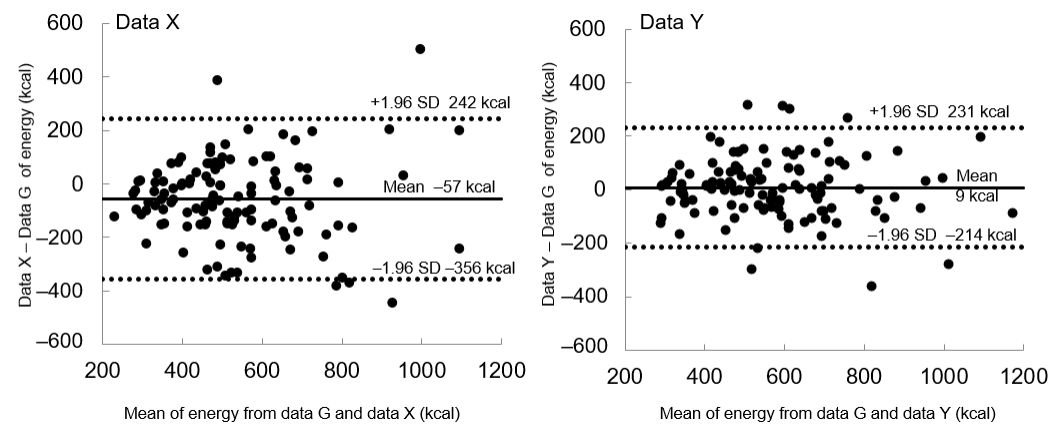
The Bland-Altman plots for energy. Data X are automatically recognized, data Y are manually adjusted, and data G are the gold standard.

Figure 3 also depicts the proportions of the mean contents of data X or Y to that of data G for selected nutrient and food groups that are often employed in dietary counseling. The contents of data Y were relatively well estimated for sample meals compared to those of data X. We depicted the Bland-Altman plots for energy (Figure 4) and macronutrients (Multimedia Appendix 1) to evaluate the agreement of the values estimated by the app and the gold standards. Both data X and Y showed acceptable agreement with data G.

**Table 3:** Nutrient and food group contents in sample meals (G), automatically estimated meals (X), and manually adjusted meals (Y) from the *CALO mama* app.

Dietary variables		Unit	Sample meals (N=120)	Automatically estimated meals (N=120)				Manually adjusted meals (N=120)		
			Mean (SD)	Mean (SD)	Difference^a^	*P*^b^ value	Mean (SD)		Difference^a^	*P*^b^ value
Weight		g	524 (129)	521 (156)	–4	.72	572 (169)		48	<.001
Energy		kcal	562 (191)	505 (189)	–57	<.001	571 (185)		9	.40
**Nutrient**										
	Protein	g	23.1 (8.9)	19.5 (8.9)	–3.5	<.001	22.6 (9.0)		–0.4	.40
	Fat	g	18.6 (11.3)	16.7 (10.9)	–1.9	.02	19.1 (10.9)		0.5	.43
	Saturated fat	g	5.41 (3.93)	4.91 (3.84)	–0.50	.10	5.56 (4.03)		0.16	.50
	n-6 polyunsaturated fat	g	3.22 (1.84)	2.49 (1.66)	–0.73	<.001	3.15 (1.88)		–0.07	.55
	n-3 polyunsaturated fat	g	0.60 (0.56)	0.48 (0.43)	–0.13	.01	0.62 (0.50)		0.01	.68
	Cholesterol	mg	126 (97)	106 (91)	–20	.001	128 (107)		2	.74
	Carbohydrate	g	68.9 (23.4)	64.6 (24.5)	–4.3	.02	70.4 (23.2)		1.5	.27
	Dietary fiber	g	4.5 (1.9)	4.5 (1.8)	0.1	.60	4.7 (1.9)		0.2	.07
	Vitamin A	µg RAE	169 (235)	140 (142)	–29	.12	173 (228)		4	.65
	Vitamin D	µg	2.4 (5.5)	1.8 (5.5)	–0.6	.19	2.1 (5.7)		–0.3	.47
	α-tocopherol	mg	2.3 (1.3)	2.0 (1.2)	–0.3	.001	2.4 (1.3)		0.1	.24
	Vitamin K	µg	76 (62)	75 (55)	–2	.65	80 (56)		4	.29
	Vitamin B1	mg	0.35 (0.23)	0.29 (0.17)	–0.06	<.001	0.35 (0.22)		0.00004	>.99
	Vitamin B2	mg	0.4 (0.21)	0.3 (0.16)	–0.03	.08	0.4 (0.17)		0.01	.52
	Niacin	mg NE	4.8 (3.2)	4.2 (2.9)	–0.6	.007	5.0 (3.1)		0.1	.44
	Vitamin B6	mg	0.40 (0.20)	0.35 (0.21)	–0.05	<.001	0.42 (0.21)		0.02	.18
	Vitamin B12	µg	1.8 (3.3)	1.4 (2.4)	–0.4	.12	1.8 (2.5)		–0.03	.86
	Folate	µg	113 (79)	105 (64)	–8	.09	118 (65)		5	.29
	Pantothenic acid	mg	1.87 (0.68)	1.63 (0.64)	–0.24	<.001	1.89 (0.63)		0.02	.66
	Vitamin C	mg	34 (26)	36 (29)	1	.37	39 (29)		5	<.001
	Sodium	mg	1166 (629)	1008 (582)	–157	.003	1241 (707)		76	.06
	Potassium	mg	734 (258)	674 (253)	–60	<.001	764 (266)		30	.08
	Calcium	mg	143 (94)	136 (95)	–6	.38	149 (92)		6	.24
	Magnesium	mg	81 (42)	70 (29)	–11	<.001	79 (34)		–2	.32
	Phosphorus	mg	323 (114)	288 (128)	–35	<.001	329 (123)		7	.38
	Iron	mg	2.3 (1.0)	2.1 (0.8)	–0.2	<.001	2.4 (0.9)		0.04	.42
	Zinc	mg	2.6 (1.0)	2.3 (1.0)	–0.3	<.001	2.6 (0.9)		–0.01	.85
	Copper	mg	0.37 (0.28)	0.30 (0.13)	–0.06	.001	0.35 (0.16)		–0.02	.23
	Manganese	mg	0.79 (0.38)	0.75 (0.34)	–0.04	.14	0.83 (0.35)		0.04	.08
	Salt	g	3.0 (1.6)	2.5 (1.5)	–0.5	<.001	3.1 (1.8)		0.2	.14
**Food group**										
	Cereals	g	132.0 (79.5)	110.2 (80.6)	–21.8	<.001	125.2 (81.0)		–6.8	.12
	Potatoes	g	9.2 (25.9)	6.1 (19.9)	–3.1	.02	9.1 (23.2)		–0.1	.95
	Pulses	g	14.8 (37.9	9.7 (24.6)	–5.1	.01	11.1 (28.6)		–3.7	.009
	Nuts	g	0.1 (0.5)	0.04 (0.5)	–0.008	.32	0.1 (0.5)		0.02	.18
	Vegetables	g	88.8 (57.8)	84.0 (59.9)	–4.8	.29	100.1 (68.6)		11.3	.02
	Green and yellow vegetables	g	30.9 (35.5)	31.6 (41.1)	0.7	.64	33.5 (39.5)		2.6	.08
	Fruits	g	21.8 (41.1)	15.3 (26.6)	–6.5	.003	15.3 (27.0)		–6.5	<.001
	Mushrooms	g	3.1 (10.1)	2.0 (9.6)	–1.1	.03	2.5 (9.8)		–0.6	.23
	Seaweeds	g	4.9 (11.6)	3.5 (9.5)	–1.4	.02	4.3 (10.5)		–0.6	.13
	Fish and shellfish	g	17.9 (38.5)	10.1 (26.1)	–7.9	.005	15.9 (34.0)		–2.0	.18
	Meats	g	30.8 (39.5)	18.9 (29.9)	–11.9	<.001	24.8 (31.3)		–6.1	.001
	Eggs	g	16.4 (22.8)	12.9 (18.9)	–3.5	.007	15.6 (23.3)		–0.9	.41
	Dairy products	g	26.5 (54.0)	31.9 (62.4)	5.4	.03	28.4 (59.5		1.9	.24
	Confectioneries	g	7.8 (23.8)	24.3 (83.3)	16.6	.02	19.0 (66.2)		11.2	.03
	Alcoholic beverages	g	2.5 (5.8)	1.6 (4.5)	–0.9	.08	2.5 (5.9)		0.0	.95

^a^Mean values of X–G or Y–G.

^b^Paired 2-sided *t* test.

## Discussion

This study examined the validity of weight, energy, 30 nutrients, and 15 food group contents estimated by the *CALO mama* app by comparing sample meals as a reference. The mean nutrient and food group contents estimated by *CALO mama* using manually adjusted data were close to those of sample meals. Most of the mean nutrient and food group contents that *CALO mama* automatically estimated were significantly lower than those of the sample meals. Nevertheless, the automatically estimated data hit the reference data in 11 out of 30 cases for nutrients and in 4 out of 15 cases for food groups without manual adjustment. These results may indicate that *CALO mama* has the potential to estimate representative intakes among populations by using an automated picture recognition system. Further, the manually adjusted data matched the reference data in 29 out of 30 cases for nutrients and 10 out of 15 cases for food groups. The estimation was even more accurate when the automatically calculated data were manually adjusted.

Many new dietary assessment tools using new technology, such as smartphones and image analysis systems, have recently been developed [6,24,25]. Although these new tools offer a wide range of feasible options to enable dietary assessment to be incorporated into daily routines [24], adequate validation studies have not yet been conducted with regard to the estimation of comprehensive nutrient and food intake. Furthermore, a study design for examining the validity of these new tools has not yet been established. Some experimental studies have evaluated the validity of predictions by using image recognition technologies embedded in mobile devices and examined whether they precisely estimate energy or a single nutrient content for sample meals. Six et al [11] examined the accuracy of the energy and protein content of prepared meals and snacks estimated by a mobile phone food recording system in 1 day among 15 adolescents by comparing duplicated meals and snacks. For many of the full-meal items, the energy and protein values estimated by the tool were accurate within ±10% of the gold standard. In terms of the intake of each participant on a single research day, many of the participants had energy values within ±10% errors and values for protein within ±20% errors. Rhyner et al [13] examined the difference between the carbohydrate content of prepared meals in a hospital and the values estimated by the mobile phone–based system over 10 days in the cases of 19 adult volunteers with type 1 diabetes. The mean error was 26.2%. Other studies have examined the accuracy of the portion size of dish items [14,15]. Although all of these experimental validation studies concluded that these new dietary assessment tools are useful and can assist in dietary assessments, the validity of nutrient or food content estimations as concluded by automated image recognition systems has not yet been examined.

Some epidemiological studies have examined the validity of dietary intakes estimated by the new tools by using a traditional study design, in which dietary intakes estimated from the new assessment tools were compared with those from a doubly labeled water method or 24-hour recall. Some studies have examined the validity of energy intake alone [16,17,19], and 1 study examined the validity of energy intake and 4 types of food [20]. Other studies have examined the validity of the intake of energy and some selected nutrients [18,19]. Many of these epidemiological validation studies showed that the dietary values from the new tools in 3- to 7-day assessments were acceptable [17-19] for assessing dietary habits among individual participants, although 1 study concluded that a 1-day assessment was inadequate [20]. The validity of these tools for estimating nutrient and food intake at the population level has not yet been adequately examined.

To the best of our knowledge, this study is the first validation study to evaluate the ability of a smartphone app with an image recognition system to estimate comprehensive nutrient and food group contents in terms of the ability to estimate mean dietary contents in over 100 meals. Five commercial diet-tracking mobile apps were recently evaluated in Japan with regard to their ability to estimate energy and nutrient intake. Only the nutrition calculation software aspect was evaluated; however, the validity, including the discrimination between dishes and the estimation of portion size by automatic image recognition, was not evaluated [26]. Our research is novel in this respect. Although the validity of nutrient and food group contents at the individual level was not adequately explored, as this study is experimental, the mean dietary values of 120 meals estimated from the *CALO mama* app were close to those included in the sample meals for many nutrients and foods.

Misreporting is inevitable in traditional self-reporting dietary assessment methods, such as dietary records, 24-hour recalls, and food frequency questionnaires. Many studies have shown that the energy intake of populations assessed by self-reporting dietary assessment methods was misreported in the range of approximately –40% to 20% compared to the doubly labeled water method [2]. Other nutrients are also assumed to be underreported or overreported to a similar extent in self-reported dietary assessments. This study showed that the proportion of the difference in nutrient contents from data X to the sample meals was –25% to 4% and that from data Y was –11% to 13%. Although we could not compare our results to those of previous traditional validation studies because of the differing study designs, our results indicate that the image recognition system may have the potential to estimate nutrient intakes among populations to the same extent as traditional dietary assessment tools. In addition, *CALO mama* immediately provides individuals with data on their dietary intake, which can be used as is or easily adjusted if needed. Compared to traditional self-reporting methods, registration, and assessments for dietary intake, the app is much lower in cost and reduces the burden on both those who make assessments and those who need to be assessed. These findings support the idea that, for many nutrients and food groups, apps with an image recognition system have great potential to estimate dietary intake. Further validation studies at both the individual and population levels are needed to confirm the accuracy of the estimations as a dietary assessment tool in large-scale epidemiological studies.

The major strength of this study is that this validity assessment examined comprehensive nutrient and food group contents, and the estimation of nutrients and food groups was made by a health app that employed an image recognition system that enables the automatic calculation of the nutrient and food content in a meal.

Several limitations of this study require mentioning. First, the generalizability of our findings is lacking because we examined the performance of a specific app, *CALO mama*. The performance of image recognition could be better or worse than our findings, depending on the app. Nevertheless, we found that manual modification can improve the accuracy of predictions carried out using image recognition. Second, not all research staff were familiar with the *CALO mama* app. Furthermore, in the protocol of this study, the research staff could modify the outputs from the app only once without eating or tasting the sample meals. However, the app has a function allowing users to modify meals after the initial registry, and they can thus correct and register their meals more precisely after eating. In this study, meal registration correction may have been inadequate. However, the results showed that most of the mean nutrient and food group contents estimated by the image recognition system were similar to the reference, especially when manually adjusted. This result may indicate that even users who are unfamiliar with the app with image recognition can have their diets assessed correctly. If users are familiar with the app and register their meals after eating, the estimation may be more precise. Third, the image recognition system has difficulty distinguishing foods with similar shapes and colors. However, it was improved by updating its prediction model and expanding the training data. There is also a plan for implementing collaborative filtering into the system; collaborative filtering will help the system to provide more precise predictions based on the combination of food items (for instance, brown liquid that comes with a Japanese meal is more likely to be miso soup rather than coffee with milk). Finally, this study examined the ability to estimate the mean values of the nutrient and food group contents of 120 meals by using the *CALO mama* app. Although the estimated values were acceptable, it cannot be concluded from the results that *CALO mama* can estimate dietary intake at the population level in daily life. Further validation studies at both the population and individual levels are needed in the epidemiological field that uses *CALO mama* as a health administration app for individuals or as a dietary assessment tool.

In conclusion, this study showed that the mean values of the nutrient and food group contents of 120 meals derived from the image recognition system in the *CALO mama* app were well estimated compared to those of the sample meals. Automatically estimated data have a certain amount of accuracy with regard to estimating nutrient and food group contents, but this accuracy is enhanced when these data are manually adjusted. Health apps embedding image recognition have the potential to contribute to “precision nutrition,” a comprehensive and dynamic approach to developing tailored nutritional recommendations in consideration of genetics, dietary habits, eating patterns, and physical activity [27,28]. They can overcome the limitations of conventional measurements, and real-time data from them will enable researchers to study how diet affects health and diseases more accurately and provide helpful dietary recommendations. Further validation studies at both the population and individual levels are essential if we are to utilize image recognition as a health administration app for individuals or as a dietary assessment tool in research.

## Appendix

Multimedia Appendix 1 The Bland-Altman plots for macronutrients. Data X are automatically recognized, data Y are manually adjusted, and data G are the gold standard.
