# Supplementary material for: Nutrient and Food Group Prediction as Orchestrated by an Automated Image Recognition System in a Smartphone App (CALO mama): Validation Study
Source: JMIR Form Res. 2022 Jan 10;6(1):e31875. doi: 10.2196/31875 (PMC8787663; doi:10.2196/31875)
Supplement: Multimedia Appendix 1 [file formative_v6i1e31875_app1.docx]

**Multimedia Appendix 1**

**Nutrient and Food Group Prediction as Orchestrated by an Automated Image Recognition System in a Smartphone App (*CALO mama*): Validation Study**

**Figures.** The Bland-Altman Plots for Macronutrients. Data X are automatically recognized, data Y are manually adjusted, and data G are the gold standard.

**
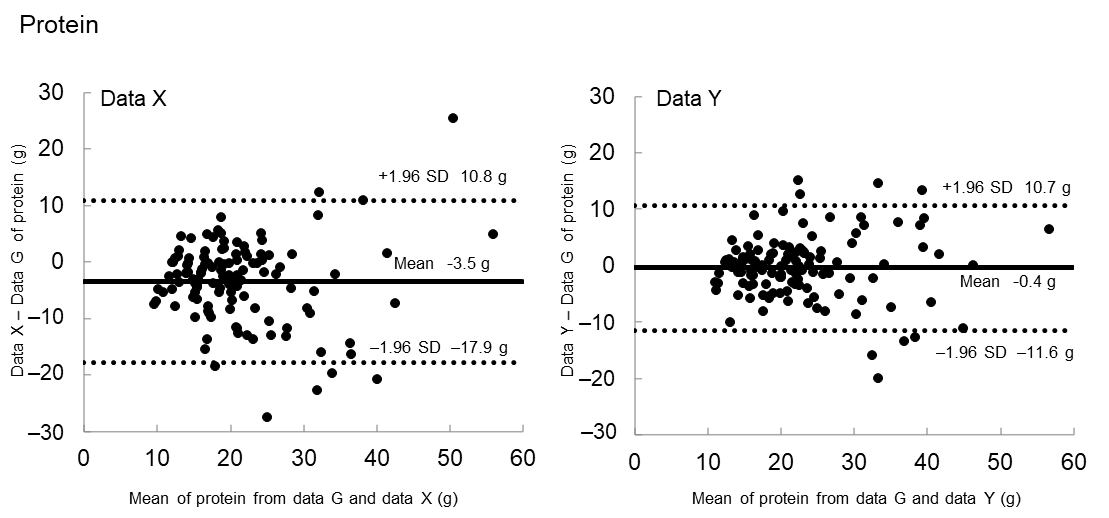
**

**
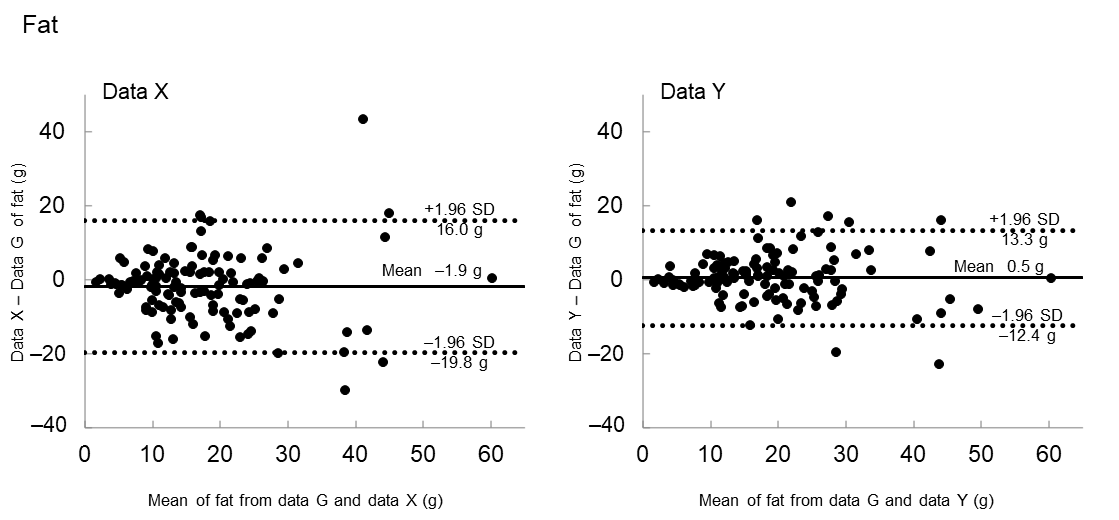
**

**
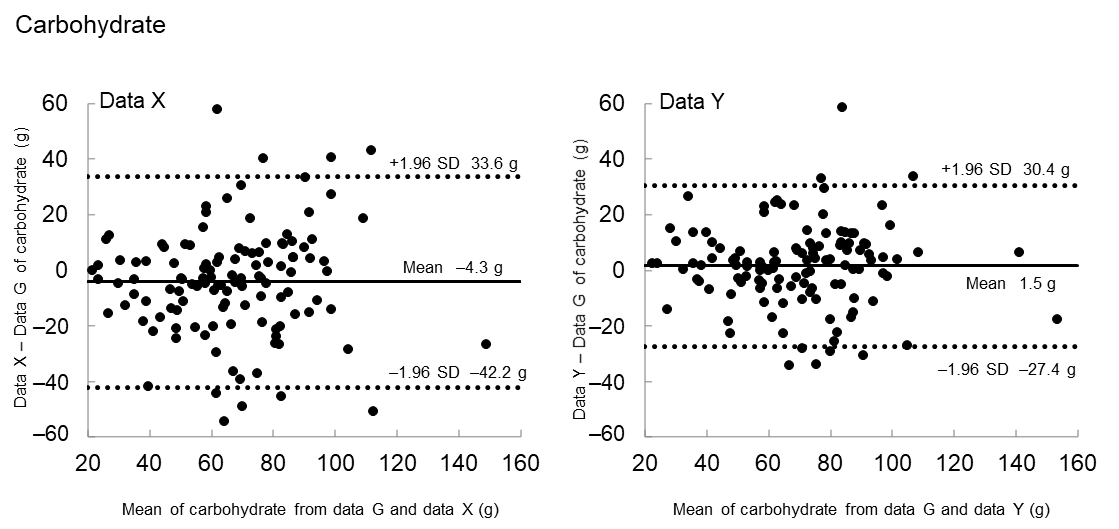
**
